# Cross-Sectional Associations Between Mediterranean Diet Adherence, Physical Activity, Satisfaction with Physical Education, and Bicycle Use Among Primary School Children

**DOI:** 10.3390/nu18030497

**Published:** 2026-02-02

**Authors:** Guillermo Moreno-Rosa, Silvia San Román-Mata, Carmen África del Pino-Morales, Manuel Castro-Sánchez

**Affiliations:** 1 Department of Didactics of Musical, Plastic and Corporal Expression, Faculty of Education and Sports Sciences of Melilla, University of Granada, 52017 Melilla, Spain; gmoreno@ugr.es; 2 Department of Nursing, Faculty of Health Sciences of Melilla, University of Granada, 52071 Melilla, Spain; silviasanroman@ugr.es; 3 Spanish Ministry of Education, Vocational Training and Sports, Provincial Directorate of Education in Melilla, 52001 Melilla, Spain; carmenafrica.delpino@edumelilla.es

**Keywords:** Mediterranean diet, physical education, out-of-school physical activity, KIDMED, satisfaction, bicycle use, school children

## Abstract

Background/Objectives: This descriptive cross-sectional study examined adherence to the Mediterranean diet (MD) among primary school students and its associations with out-of-school physical activity, bicycle availability and use, and satisfaction with physical education (PE). The MD is regarded as an ideal dietary pattern for promoting health during childhood and adolescence. Its well-established benefits support its integration into nutrition and health education within the PE curriculum. However, the connection between adherence to the MD and factors such as satisfaction with PE, bicycle availability and use, and out-of-school physical activity during primary education remains insufficiently explored. Methods: The study included 347 primary school students (53.6% girls; *M_age_* = 10.55, *SD* = 0.97). Data were collected using an ad hoc questionnaire including sociodemographic information, out-of-school physical activity, and bicycle availability and use. MD adherence was evaluated using the KIDMED index, while satisfaction with PE was measured with the Spanish version of the Physical Activity Class Satisfaction Questionnaire (PACSQ). Results: No significant associations were found between MD adherence and out-of-school physical activity (*χ*^2^ = 0.882; *p* = 0.663) or bicycle use (*χ*^2^ = 4.767; *p* = 0.092). In contrast, a significant association was observed between MD adherence and satisfaction with PE (*p* < 0.002), including most of its dimensions. Conclusions: Overall, the findings indicate an association between satisfaction with PE and MD adherence, which should be interpreted as exploratory and non-causal in nature.

## 1. Introduction

In most European countries, physical education (PE) is a compulsory subject in the curriculum and is taught at different educational levels [1]. Its main objectives include the comprehensive development of individuals across multiple domains—physical, cognitive, emotional, and social [2]—as well as the promotion of healthy habits and lifestyles [3,4], including adherence to the Mediterranean diet (MD). Consequently, health is a key component of PE, given the preventive role of physical activity in relation to sedentary behaviour [5,6], obesity [7,8], childhood diabetes [9], and the risk of cardiovascular disease in adulthood [10,11]. In relation to the ALADINO study [12], obesity in children and adolescents is associated not only with genetic factors but also with poor eating habits—such as abandoning the MD [13]—and increased sedentary behaviours in daily life [14,15], which disregards the World Health Organization (WHO) recommendations to engage in 60 min of moderate-to-vigorous daily physical activity for people aged 6 to 16 [16]. Moreover, for many children and adolescents, the only physical activity they engage in each week takes place during PE classes [17], during which the time spent in motor engagement and moderate-to-vigorous physical activity accounts for less than one-third of the total session time [18].

Despite the limited time available, the PE curriculum in primary education in Spain is oriented towards developing health-related skills through interventions aimed at promoting habits associated with healthy physical activity, as well as fostering critical attitudes among pupils towards harmful or unhealthy behaviours [19]. Therefore, it is a priority for PE teachers to promote daily physical activity [20,21] from early childhood [22], encourage students to adopt a critical perspective on the consequences of a sedentary lifestyle [23], and raise awareness of the importance and benefits of following healthy eating patterns, including the MD [24]. Such actions also involve promoting a critical attitude towards unhealthy dietary habits, such as the regular consumption of ultra-processed foods high in fats and sugars [14,25], as well as the intake of energy drinks, whose presence in children’s diets constitutes a significant health risk factor [26].

The MD, characterized by a high intake of fresh and seasonal foods—particularly fruits, vegetables, legumes, nuts, and cereals—with minimal processing, predominant use of olive oil, and moderate consumption of dairy products, mainly yogurt and cheese [24,27,28], has been associated with better body composition and a lower risk of overweight and obesity [29], improvements in anti-inflammatory biomarkers [24], enhanced psychological and emotional well-being [30], and the adoption of active lifestyle habits. The MD has also been shown to constitute a dietary model of great sociocultural value, as its low environmental impact contributes to the conservation of biodiversity and the promotion of local economies [27,28]. Consequently, PE should not only focus on promoting physical activity but should also become a key setting for nutrition education [13,31], making it an optimal tool for health promotion [32] and a priority for educational authorities [33,34].

However, despite the wide range of opportunities offered by PE, achieving the objectives set out in the curriculum is not an easy task, especially when considering students’ perceptions of their experiences in PE classes, which are not entirely positive [3,35,36]. Children’s satisfaction with PE has been studied from various theoretical approaches, including self-determination theory (SDT), achievement goal theory (AGT), and meaningful PE (MPE) [37]. According to SDT [38], students differ in their levels of motivation. Specifically, students with low self-determined motivation participate in PE mainly because of anticipated consequences, such as obtaining a reward or avoiding punishment [37]. In contrast, highly self-determined students are more engaged in class due to the interest and satisfaction they derive from the activities or the personal value they place on PE [39]. From the perspective of AGT [40], differences in children’s achievement goal orientation translate into differences in PE-related outcomes, such as enjoyment and participation [41]. Ego-oriented children focus on outperforming others and demonstrating competence based on normative standards. In contrast, task-oriented students focus on learning, enjoying PE, cooperating with others, feeling satisfied, persisting in tasks [42,43], and showing high levels of participation [44]. These motivational differences may influence students’ engagement with PE and, consequently, their health-related behaviours, including dietary habits such as adherence to the MD.

In the search for experiences that spark interest, satisfaction and adherence to physical activity, cycling is worth highlighting, as its benefits at different stages of life are well documented in the scientific literature [45]. It is positively associated with higher levels of physical activity and improved health at all ages [46], especially in children and adolescents [47]. Cycling increases the time spent in moderate-to-vigorous physical activity in children, as recommended by the WHO [48], and helps maintain long-term physical activity habits [49]. Frequent cycling is also associated with a reduced risk of cardiovascular disease [50], which contributes to lower cholesterol levels and improved glucose metabolism [51]. From an energy perspective, cycling involves more intense physical activity than walking [46], thereby enhancing physical fitness [52]. Within the school-aged population, the previous benefits provide a comprehensive framework for understanding the significance of cycling as a form of physical activity. In this regard, promoting the recreational use of bicycles, as well as the use as a form of active mobility in children, may help them acquire and practice skills that contribute to the development of healthy lifestyle habits and improved physical fitness [1].

Despite the existing evidence on the benefits of physical activity, adherence to the MD, cycling, and satisfaction with PE, no studies to date have examined the relationships among these factors in the school context, and more specifically in primary education. This gap in knowledge limits a comprehensive understanding of how these factors interact, preventing health and PE professionals from designing and implementing more holistic and effective educational interventions to promote healthy lifestyles. Based on this background, the present study hypothesises that (H1) higher satisfaction with PE is associated with greater adherence to the MD; (H2) higher out-of-school physical activity is associated with greater adherence to the MD; and (H3) bicycle availability and use are associated with greater adherence to the MD. Therefore, this study aims to analyse adherence to the MD and its relationship with physical activity outside school, satisfaction with PE, and bicycle ownership and use in primary school children.

## 2. Materials and Methods

### 2.1. Study Design and Participants

This study employed a descriptive and comparative cross-sectional design, which included a total of 347 students of both sexes (46.4% boys, 53.6% girls) aged 9 to 13 years (*M_age_* = 10.55, *SD* = 0.97), recruited from two public primary schools in a Spanish autonomous city located in North Africa. Participants were selected using a convenience sampling method. The sample represents approximately 9.47% of the total students’ population in the last three years of primary education in the city. However, due to the non-probabilistic nature of the sampling procedure, the findings should be interpreted with caution in terms of external validity. Inclusion criteria were enrolment in the 4th, 5th, or 6th grade of primary school, consent from legal guardians, and the absence of any diagnosed neurodevelopmental disorder.

The schools that participated in the study are publicly funded. Both schools were located in different neighbourhoods of the Autonomous City and shared similar socio-economic and educational characteristics. Most students were Spanish nationals of Maghreb origin, from families with low to medium-low incomes. The majority of parents worked in the service sector, including self-employed workers, construction workers, cleaning staff, and military personnel, although there was a high rate of unemployment in some families. From an educational perspective, both schools faced significant challenges, as their students consistently achieved low scores on the annual diagnostic assessments conducted by the Ministry of Education, which evaluate competencies in reading comprehension, arithmetic, problem-solving, and knowledge of natural and social sciences.

### 2.2. Variables and Instruments

#### 2.2.1. Sociodemographic Data, Physical Activity Outside School, and Bicycle Ownership and Use

An ad hoc questionnaire was used to collect information on participants’ date of birth, sex, and school grade. These variables were considered descriptive characteristics of the sample and potential contextual factors. In addition, participants were asked about their engagement in physical activity outside the school environment (“Do you usually engage in physical activity outside of school?”) and about bicycle ownership and use (“Do you have a bicycle at home, and do you usually use it?”). Both questions were answered dichotomously (“Yes” or “No”). Each item was included as a brief indicator to ensure comprehension and feasibility in a primary school population.

#### 2.2.2. MD Adherence

The KIDMED questionnaire (Mediterranean Diet Quality Index for children and adolescents), developed by Serra-Majem et al. [53], assesses adherence to the MD through 16 dichotomous items (Yes/No). Of these, 12 items reflect positive dietary patterns, such as the consumption of fruits, vegetables, fresh produce, legumes, cereals, fish, and olive oil, while the remaining four items assess negative dietary patterns, such as skipping breakfast, consuming pastries or sweets, or eating at fast-food restaurants. Favourable items are scored +1 and unfavourable items −1. The total score is calculated as the sum of all responses, ranging from 0 to 12, with adherence levels classified as poor (0–3), fair (4–7), and good (8–12). Due to its simplicity, KIDMED has been widely used to assess adherence to the MD in children and adolescents. The reliability of the original instrument showed α = 0.854, while in the present study, a coefficient of α = 0.712 was obtained, indicating acceptable internal consistency. This result is consistent with previous studies and reflects the nature of KIDMED as an index of heterogeneous dietary habits with dichotomous items, rather than a unidimensional scale.

#### 2.2.3. Satisfaction with PE Classes

The Spanish version of the Physical Activity Class Satisfaction Questionnaire (PACSQ), adapted and validated by Sicilia et al. [54], was used to assess satisfaction with PE classes. The PACSQ is a valid and reliable instrument for measuring satisfaction with PE in a multidimensional manner. It consists of 33 items distributed across nine dimensions, including aspects such as satisfaction with teaching, relaxation, cognitive development, fun and enjoyment, and improvement of physical fitness and health, among others. Each item is rated on an 8-point Likert scale, where 1 indicates “completely dissatisfied” and 8 “completely satisfied” with the PE classes attended. Scores are calculated by summing the items corresponding to each dimension, as well as a total score reflecting overall satisfaction with PE. The original validation study confirmed a nine-factor first-order factor structure underlying a second-order factor labelled “satisfaction,” showing acceptable fit indices (*χ*^2^/df = 4.55; CFI = 0.93; IFI = 0.93; RMSEA = 0.064; SRMR = 0.044), supporting the construct validity of the instrument in its Spanish version. In the present study, the questionnaire demonstrated excellent internal consistency, with a Cronbach’s alpha of *α* = 0.933 for the total score, indicating excellent reliability in our sample.

### 2.3. Procedures

Authorisation to conduct the research was obtained from the competent educational authority of the Autonomous City (Provincial Directorate of the Ministry of Education), which approved its implementation (registration number 201708380). Following this approval, the researchers contacted the headteachers and PE teachers of both schools to explain the study’s objectives and procedures and to obtain their consent for participation. In addition, informed consent was obtained from the legal guardians of each pupil, as all participants were minors.

After receiving the necessary authorisations, the questionnaires were administered in a regular classroom during PE class time. PE teachers were responsible for administering and explaining the questionnaires, following standardised instructions provided by the research team. Researchers were present to verify that the process was carried out correctly and to answer any questions that arose during data collection. The questionnaires were completed anonymously by the participants. The average duration of questionnaire completion was approximately 35–40 min. The anonymity of the participants was guaranteed at all times, and they were informed that the records would be used solely for scientific purposes. Of the 367 questionnaires submitted, 347 were valid, with a total of 20 being discarded because they were incomplete or incorrectly completed. Once data collection was complete, the researchers sent a letter of thanks to the headteacher and PE teachers, undertaking to share the results obtained for informational purposes only, while always guaranteeing the confidentiality of the students.

This study has been approved by the University Ethics Committee (register number 530/CEIH/2018) and adheres to the principles established in the Declaration of Helsinki.

### 2.4. Statistical Analysis

Statistical analyses were conducted using IBM SPSS Statistics 24.0 (IBM Corp., Armonk, NY, USA). Descriptive statistics were calculated for all study variables, including adherence to the MD (KIDMED score), satisfaction with PE (PACSQ total and subscale scores), physical activity outside school, and bicycle ownership and use, using means, standard deviations, frequencies, and percentages. Relational analyses were performed to examine associations between variables. Differences in continuous variables (e.g., KIDMED and PACSQ scores) between groups were analysed using Student’s *t*-test or one-way ANOVA, as appropriate.

Normality of the data was assessed using skewness and kurtosis values for each questionnaire item, with values below 2 considered acceptable for normality in social science research involving child samples, supporting the use of parametric tests. Internal reliability of the instruments was evaluated using Cronbach’s alpha, with a confidence level of 95%.

For dichotomous variables such as physical activity outside school and bicycle ownership/use, frequencies and percentages were reported, and associations with categorical variables were analysed using chi-square tests. Given the exploratory nature of the study, no multivariable models were applied to control for potential confounding variables (e.g., age, grade, or socioeconomic context), which is acknowledged as a limitation. On the other hand, considering the convenience sampling and exploratory design, the findings should be interpreted cautiously in terms of external validity and generalisation.

## 3. Results

### 3.1. Descriptive Analysis of Variables

Table 1 presents the results of the descriptive analysis conducted on the 347 participants in the study. Of the total sample, 45.8% (*n* = 159) were boys, while 54.2% (*n* = 188) were girls.

Regarding engagement in physical activity outside school, 71.2% of students (*n* = 247) reported regularly participating in some form of physical activity, whereas 28.8% (*n* = 100) reported not doing so. Similarly, the majority of participants (*n* = 262; 75.5%) reported having access to a bicycle at home and using it regularly.

In terms of MD adherence, nearly 69.7% of students were classified as needing to improve their dietary habits (15.3% with a poor-quality diet and 54.5% needing improvement), while 30.3% followed an optimal diet.

Participants also reported high levels of satisfaction with PE, with scores approaching 6 points across all related dimensions. The highest-scoring dimensions, indicating greater satisfaction with PE, were cognitive development (*M* = 6.31), mastery experiences (*M* = 6.24), fun and enjoyment (*M* = 6.23), teaching (*M* = 6.18), and health improvement (*M* = 6.16), among others (Table 2).

### 3.2. Comparative Analysis of Physical Activity Outside School, Bicycle Ownership and Use, MD Adherence, and Satisfaction with PE by Sex

In the analysis of physical activity outside school by sex, the data revealed statistically significant differences (*p* = 0.009), with boys reporting higher levels of physical activity (78.3%; *n* = 126) compared to girls (65.1%; *n* = 121). Similarly, statistically significant differences were found in bicycle ownership and use by sex (*p* = 0.045), with a higher percentage of boys having and using a bicycle (80.7%; *n* = 130) compared to girls (71.0%; *n* = 132). Regarding the MD adherence, no significant differences were observed between boys and girls, suggesting that diet adherence was independent of sex in this study (Table 3).

In the analysis of satisfaction with PE by sex, no statistically significant differences were found for most of the study variables, indicating similar levels of satisfaction between boys and girls. However, two non-significant trends were observed: girls scored higher in the health improvement dimension (*M* = 6.28 vs. *M* = 6.01), whereas boys scored higher in normative success (*M* = 5.79 vs. *M* = 5.49). Of particular interest, the only statistically significant difference was observed in the “mastery experiences” dimension, where girls reported a higher perception of competence and mastery (*M* = 6.40) compared to boys (*M* = 6.05), as shown in Table 4.

### 3.3. Comparative Analysis of MD Adherence by Physical Activity Outside School, Bicycle Ownership and Use, and Satisfaction with PE

When analysing physical activity outside school in relation to MD adherence, no statistically significant association was found (*χ*^2^ = 0.882; *p* = 0.663), indicating that, in this sample, engagement in out-of-school physical activity and adherence to healthy dietary patterns appeared to be independent behaviours. Although a slight trend was observed towards a more optimal diet among physically active participants, this difference was not statistically significant (Table 5).

Regarding bicycle ownership and use in relation to MD adherence, participants who had and used a bicycle showed slightly higher dietary adherence scores. However, the analysed data indicated no statistically significant association between these variables in the sample (*χ*^2^ = 4.767; *p* = 0.092).

In the analysis of MD adherence in relation to students’ satisfaction with PE, results showed statistically significant associations for most PE dimensions, except “interaction with others” and “normative success”. Specifically, PE overall satisfaction was significantly associated with higher MD adherence (*p* = 0.002), indicating that greater MD adherence corresponded to higher PE overall satisfaction.

Regarding the “teaching perception” dimension, scores progressively increased with better diet quality (*p* = 0.001), indicating that students with an optimal diet rated the quality of teaching more highly. Similarly, the “relaxation” dimension (*p* < 0.001) showed a notable improvement, with students following an optimal diet feeling more relaxed during PE sessions. Likewise, an optimal diet was associated with higher perceived cognitive benefits in PE (*p* = 0.001), health and fitness improvement (*p* = 0.009), and mastery experiences (*p* = 0.017) dimensions. These outcomes suggest that students with better diet quality perceived greater health benefits from PE while simultaneously feeling more competent. Similarly, scores in the fun and enjoyment (*p* = 0.038) and recreational experiences (*p* = 0.010) dimensions increased with better diet quality, indicating that an optimal diet contributed to a greater sense of enjoyment during PE sessions (Table 6).

Although several statistically significant associations were observed between MD adherence and dimensions of satisfaction with PE, these differences should be interpreted cautiously, as the magnitude of the observed differences appears modest and may have limited practical relevance. Accordingly, the results are presented as indicative associations rather than strong effects.

**Figure 1:**
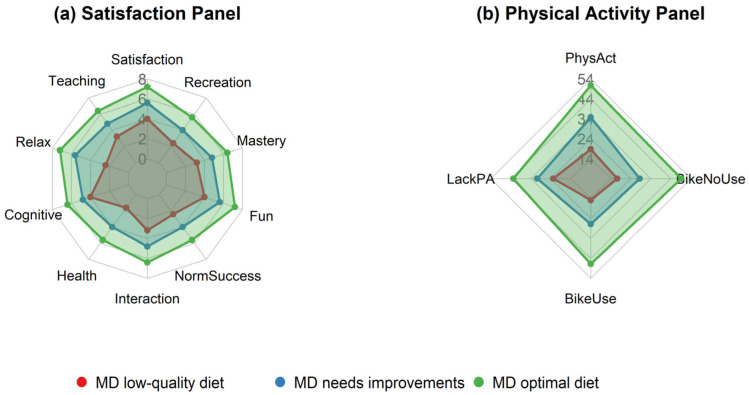
Multidimensional profiles associated with MD quality according to (**a**) PE satisfaction and (**b**) out-of-school physical activity, and bicycle ownership and use.

The general patterns observed regarding the variation in satisfaction with PE, out-of-school physical activity and bicycle use according to the three levels of adherence to the MD (optimal, needs improvement and low-quality diet) are presented in Figure 1.

## 4. Discussion

The purpose of this study was to assess adherence to the MD among a sample of primary school students and to examine its association with out-of-school physical activity, bicycle ownership and use, and satisfaction with PE. The results showed no statistically significant associations between MD adherence and out-of-school physical activity or bicycle ownership and use. In contrast, a statistically significant association was observed between adherence to dietary patterns characteristic of the MD and students’ overall satisfaction with PE, as well as with most of its component dimensions.

Descriptive analyses indicated a high prevalence of physical activity among the participants, with higher levels observed among boys. Approximately 80% of boys reported being physically active, compared with 60% of girls who indicated participation in some form of extracurricular physical activity. These findings are consistent with those reported by Román et al. [55], who observed that Spanish girls are generally less physically active than boys, although they do not exhibit a high prevalence of sedentary behaviours. Similarly, the present results align with previous studies showing lower levels of physical activity among girls compared to their male counterparts [14,56,57]. The lower engagement in out-of-school physical activity observed among girls may be related to a greater amount of time spent in sedentary activities, such as studying, household tasks, listening to music, or reading, as well as to differences in activity preferences, with girls more frequently engaging in activities such as dance [55]. In contrast, boys tend to show a greater orientation towards physical activity, which has been associated with a higher perceived motor competence and a stronger preference for competitive sports-based activities [58]. All these explanatory factors were not directly examined in the present study and should therefore be interpreted cautiously.

Regarding bicycle ownership and use, most participants reported having access to a bicycle and using it with some regularity, with a higher prevalence observed among boys than girls. This pattern is consistent with previous research showing more frequent bicycle use among boys compared to girls [59,60,61,62]. In line with Bell et al. [59], these findings suggest that, before implementing cycling promotion or education programmes, it may be important to explore the factors underlying lower bicycle use among girls, including potential differences in interest as well as perceived barriers related to traffic safety among both children and their parents [60], for recreational and transport-related cycling. In light of these findings, future studies should empirically examine these factors to gain a better understanding of sex-related differences in cycling behaviours.

With respect to the MD adherence, approximately two-thirds of the participants showed suboptimal dietary patterns. Specifically, 15.3% presented low-quality diets, while 54.5% required improvement, and only 30.3% exhibited optimal adherence. These findings are consistent with those reported by Herrera-Ramos et al. [63], who observed a decline in MD adherence among Spanish children and adolescents over the past two decades, largely attributed to increased consumption of ultra-processed foods and frequent fast-food intake. However, the present results contrast with those of Pinel-Martínez et al. [14], who reported optimal adherence to the MD among primary school students. By sex, no statistical differences were found between boys and girls, suggesting that this variable does not appear to influence adherence to specific dietary patterns. These results are consistent with those reported by Iaccarino Idelson et al. [64], who, in their systematic review, stated that in most studies, MD adherence was not associated with the sex of children and adolescents. Likewise, in the study of Domínguez-Martín et al. [28], no interactions were found between MD adherence and sex. Therefore, adherence to a particular dietary pattern, such as the MD, does not seem to be linked to sex but rather to the result of interactions with other factors, such as socioeconomic status, food prices, culture, family behavioural patterns, or personal preferences [63], among others. Accordingly, the findings highlight new avenues for research aimed at elucidating the role of contextual influences, such as socioeconomic and family-related factors.

In terms of satisfaction with PE, most study participants reported being highly satisfied. Cognitive development was the highest-rated dimension (*M* = 6.31), followed by mastery experiences (*M* = 6.24), fun and enjoyment (*M* = 6.23), teaching quality (*M* = 6.18), and health and physical fitness improvement (*M* = 6.16), among others. These findings align with those reported by Gómez-López et al. [65], who observed a strong tendency for students to enjoy and feel satisfied with PE classes, as well as a high intention to engage in physical activity. By sex, a statistically significant difference was found only in the “mastery experiences” dimension, with girls reporting higher perceptions than boys. No associations were observed for overall PE satisfaction or for the other dimensions, indicating similar satisfaction levels across sexes. In contrast, girls tended to rate health improvement higher (*M* = 6.28), whereas boys tended to rate normative success higher (*M* = 5.79). These results are partially consistent with previous studies in secondary education [66], which reported high satisfaction among both boys and girls. On the other hand, they contrast with the findings reported by Navarro-Patón et al. [67], who observed that girls obtained lower satisfaction scores than boys in both primary and secondary education.

The statistically significant difference in the “mastery experiences” dimension among girls may reflect their greater appreciation of learning outcomes acquired throughout the PE process. Likewise, girls’ tendency to rate improvements in health and physical fitness more positively may be associated with greater health awareness and its link to emotional well-being [54]. In this context, the observed differences appear to reflect variations in perceived learning experiences within PE rather than differences in objectively assessed competence.

Concerning the comparative analysis between MD adherence and out-of-school physical activity, no statistical association was found (*χ*^2^ = 0.882; *p* = 0.663), and therefore Hypothesis 2 (H2) was not supported. In the analysed sample, physical activity outside of school and adherence to this dietary pattern appeared to be independent behaviours. However, a slight trend towards a healthier diet was observed among the more physically active participants, although the differences were not statistically significant. These findings contrast with those reported in several studies [24,39,68], which confirmed an association between MD adherence and greater engagement in physical activity. Nevertheless, previous research has also reported inconsistent or weak associations between these behaviours in child and adolescent populations, which is in line with the findings of Gascón et al. [69], who observed that higher adherence to the MD does not necessarily coincide with increased adoption of physical activity habits among children and adolescents.

The lack of a relationship between MD adherence and physical activity in the present study may be attributed to factors not directly measured, such as the high participation in extracurricular physical and sports activities among the city’s school population, as well as the socioeconomic difficulties reported in many families of the participating students. In the autonomous city where the study was conducted, extensive, attractive, and even free recreational and sports programs are available, which likely contribute to high levels of physical activity among children. However, such promotion is not accompanied by campaigns raising awareness of the health benefits of following healthy dietary patterns, such as the MD, which are essential for fostering a positive attitude towards health in children and adolescents. On the other hand, the low socioeconomic status of a significant portion of the participating students’ families is associated with less healthy eating habits. As Álvarez et al. [70] highlight, socioeconomic difficulties can constitute a significant barrier to health in children and adolescents. Based on these findings, public administrations and sports organisations with responsibilities in education and health promotion should implement campaigns to promote the MD, aiming to prevent diet-related diseases in children. These initiatives should also foster critical thinking about harmful eating habits that may contribute to the development of conditions such as diabetes and obesity in children and adolescents. It is worth noting that students in the sample generally reported high levels of physical activity; however, the intensity, duration, and characteristics of these activities were not explored. Likewise, socioeconomic information regarding the families was not collected in this study. While these contextual factors provide plausible explanations, they were not directly measured, and their influence remains to be confirmed. This highlights the value of future research incorporating direct assessments of physical activity, programme participation, and socioeconomic variables to gain a more comprehensive understanding of their relationship with dietary adherence.

On the other hand, regarding bicycle ownership and use, and their relationship with MD adherence, a trend towards higher MD adherence was observed among participants who owned and used bicycles, although these differences were not statistically significant (*χ*^2^ = 4.767; *p* = 0.092), similar to the findings for physical activity outside of school. Accordingly, Hypothesis 3 (H3) was not confirmed. While no comparable studies were identified, these results may again be explained by the lack of campaigns promoting healthy habits and active recreational mobility by bicycle. Therefore, schools and educational institutions should prioritise implementing campaigns that promote cycling and raise awareness of the benefits of following healthy dietary patterns to support overall health. Nevertheless, given the absence of statistical significance and the exploratory nature of the study, these findings should be interpreted cautiously and warrant further investigation.

In the analysis of MD adherence according to students’ satisfaction with PE, statistically significant associations were observed in most dimensions related to PE satisfaction and MD adherence, except for “interaction with others” and “normative success”. Specifically, overall satisfaction with PE was associated with higher MD adherence. Regarding the teaching perception dimension, optimal adherence to the diet was linked to progressively higher scores in this dimension, reflecting a more positive perception of teaching quality among students with an optimal diet. Moreover, these participants reported feeling more relaxed during PE sessions, resulting in higher scores in the “relaxation” dimension. Optimal MD adherence was also associated with greater perceived cognitive benefits, health and fitness improvements, as well as mastery experiences. These results indicate that students with better diet quality experienced more positive and satisfying PE classes across multiple dimensions. Importantly, these associations refer to subjective experiences rather than to objective competence, performance, or motor skill acquisition. Similarly, scores in the “fun and enjoyment” (*p* = 0.038) and “recreational experiences” (*p* = 0.010) dimensions increased as diet quality improved, indicating that an optimal diet enhanced students’ enjoyment during PE sessions.

Overall, the results of this study indicate that higher satisfaction with PE among primary school students is positively associated with greater adherence to the MD, thereby supporting Hypothesis 1 (H1). This finding is consistent with the study by Lirola et al. [13], which included 3415 secondary school students and reported an association between higher perceived satisfaction in PE classes and more favourable attitudes towards healthy eating. Nevertheless, these associations should be interpreted cautiously, as no conclusions regarding directionality can be drawn.

From an interpretative perspective, these findings suggest that satisfaction with PE and adherence to the MD may be interrelated within the school context. Rather than implying a direct educational effect, the results highlight the relevance of further longitudinal and intervention-based studies to explore whether and how positive experiences in PE are linked to dietary behaviours. In this sense, PE may represent a relevant setting for examining the coexistence of physical activity, well-being, and healthy eating habits in primary education, supporting the view that one of the main goals of sports and health professionals should be to promote participation in physical activity for health and enjoyment, rather than solely for competitive achievements [71].

Several limitations should be considered when interpreting the findings of this study. First, the descriptive, cross-sectional design prevents analysis of the temporal evolution of physical activity habits, bicycle use, and dietary patterns within the sample. It precludes any causal inference between the studied variables. Accordingly, the results should be interpreted as exploratory and associative in nature. Additionally, although the sample size was representative at the level of the autonomous city, the results cannot be generalised to national or international populations. Moreover, researcher supervision during data collection may have introduced some bias in the responses. The use of an ad hoc questionnaire with dichotomous responses to assess extracurricular physical activity, as well as bicycle ownership and use, also limits the precision in quantifying these behaviours and reduces measurement sensitivity, which may have affected the detection of variability within the sample. These brief indicators were included due to feasibility considerations and to ensure comprehension and minimise respondent burden in a primary school population. However, they cannot replace validated instruments. In addition, the statistical analyses were mainly based on unadjusted bivariate comparisons, without the use of multivariable models to control for potential confounding factors such as age, grade level, socioeconomic context, or school-level effects. This analytical approach may have limited the internal validity of the observed associations. Furthermore, effect sizes were not systematically reported, and no corrections for multiple testing were applied, which may increase the risk of type I error and reduce the robustness of the findings.

Future research should employ validated and more sensitive measures adapted to children to better capture physical activity patterns and active transportation behaviours. In this regard, future studies should consider using validated instruments to assess physical activity and designing more comprehensive questionnaires that allow for a deeper analysis of variables such as the type of bicycle used or the frequency of use per week.

From an analytical perspective, future research should incorporate multivariable statistical models to control for relevant confounding variables (e.g., age, grade, socioeconomic context, and school-level effects), systematically report effect sizes, and apply appropriate corrections for multiple testing to provide more robust and internally valid evidence.

Additionally, longitudinal or intervention-based designs would help clarify the directionality of the observed associations. It would also be valuable to examine the implementation of cycling education programs within PE and their relationship with student satisfaction and adherence to the MD, as well as to evaluate the impact of awareness campaigns aimed at promoting healthy dietary habits.

## 5. Conclusions

The main finding of this study was the observed association between adherence to the Mediterranean diet (MD) and satisfaction with physical education (PE) among primary school students. Higher levels of satisfaction with PE were associated with more favourable perceptions of the learning experience, including enjoyment, relaxation, perceived cognitive and health benefits, and mastery experiences, which co-occurred with higher MD adherence. A high prevalence of physical activity and bicycle use was observed, particularly among boys, alongside suboptimal MD adherence in a considerable proportion of students, with no significant differences by sex. Although no statistically significant associations were found between physical activity or bicycle use and MD adherence, a slight trend towards higher adherence was observed among the more physically active participants.

Taken together, these findings suggest that satisfaction with PE and adherence to the MD may be interrelated within the school context. However, given the cross-sectional design, no conclusions can be drawn regarding directionality or educational impact. Accordingly, these associations should be interpreted as exploratory and hypothesis-generating rather than indicative of a direct educational effect. In this sense, the observed associations suggest that PE may represent a relevant school context in which students’ satisfaction with PE and dietary behaviours are interrelated, highlighting the need for further longitudinal and intervention-based research to examine these relationships in greater depth. Within this exploratory framework, educational experiences that prioritise students’ satisfaction and engagement may represent a potentially effective strategy for fostering both physical and dietary health in primary school students, although this assumption requires confirmation through more robust study designs.

## Figures and Tables

**Table 1:** Descriptive analysis of the variables.

**Sex**	Boys	45.8% (*n* = 159)
	Girls	53.6% (*n* = 186)
Engagement in out-of-school physical activity	Yes	71.2% (*n* = 247)
	No	28.8% (*n* = 100)
Having and using the bicycle	Yes	75.5% (*n* = 262)
	No	24.5% (*n* = 85)
MD adherence	Low-quality diet	15.3% (*n* = 53)
	Needs improvement	54.5% (*n* = 189)
	Optimal diet	30.3% (*n* = 105)

Legend: Values are expressed as percentages (%) and absolute frequencies (*n*).

**Table 2:** Descriptive analysis of satisfaction with PE.

Satisfaction with PE		*M*	*SD*
	PE Overall Satisfaction	6.11	1.20
	Teaching	6.18	1.46
	Relaxation	5.95	1.48
	Cognitive development	6.31	1.48
	Health and Fitness Improvement	6.16	1.45
	Interaction with Others	6.08	1.47
	Normative Success	5.63	1.67
	Fun and Enjoyment	6.23	1.66
	Mastery Experiences	6.24	1.56
	Recreational Experiences	6.09	1.39

Legend: *M* = Media; *SD* = Standard deviation.

**Table 3:** Comparative analysis of physical activity outside school, bicycle ownership and use, MD Adherence, and satisfaction with PE by Sex.

		Sex		Sig.
		Boys	Girls	
Engagement in out-of-school physical activity	Yes	78.3% (*n* = 126)	65.1% (*n* = 121)	0.009 *
	No	21.7% (*n* = 35)	34.9% (*n* = 65)	
Having and using the bicycle	Yes	80.7% (*n* = 130)	71.0% (*n* = 132)	0.045 *
	No	19.3% (*n* = 31)	29.0% (*n* = 54)	
MD adherence	Low-quality diet	16.1% (*n* = 26)	14.5% (*n* = 27)	0.914
	Needs improvement	54.0% (*n* = 87)	54.8% (*n* = 102)	
	Optimal diet	29.8% (*n* = 48)	30.6% (*n* = 57)	

Legend: Values are expressed as percentages (%) and absolute frequencies (*n*). Chi-square tests were used to examine differences between boys and girls. * *p* ≤ 0.05. Abbreviations: Sig. = significance (two-tailed).

**Table 4:** Satisfaction with PE by sex.

	Sex				Levene’s Test		*t*-Test Sig. (Two-Tailed)
	Boys		Girls				
	*M*	*SD*	*M*	*SD*	*F*	Sig.	
PE Overall Satisfaction	6.04	0.09	6.18	0.08	0.140	0.907	0.314
Teaching	6.13	0.11	6.23	0.10	0.236	0.627	0.549
Relaxation	5.87	0.11	6.02	0.10	0.414	0.520	0.351
Cognitive development	6.23	0.11	6.38	0.10	0.186	0.667	0.353
Health and Fitness improvements	6.01	0.12	6.28	0.10	0.212	0.645	0.090
Interaction with others	6.04	0.11	6.10	0.10	0.018	0.893	0.708
Normative Succes	5.79	0.12	5.49	0.12	1.458	0.228	0.094
Fun and enjoyment	6.14	0.13	6.31	0.11	0.407	0.524	0.346
Mastery Experiences	6.05	0.13	6.40	0.10	2.196	0.139	0.037 *
Recreational Experiences	6.01	0.11	6.15	0.09	0.074	0.786	0.333

Legend: Differences between sexes were analysed using Student’s *t*-test for independent samples. Levene’s test for equality of variances is reported for each variable (*F* and significance). * *p* ≤ 0.05. Abbreviations: Sig. = significance (two-tailed). *M* = Media; *SD* = Standard deviation.

**Table 5:** MD Adherence by physical activity outside school and bicycle ownership and use.

		MD Adherence			Cramer’s V	Sig.
		Low-Quality Diet	Needs Improvement	Optimal Diet		
Engagement in out-of-school physical activity	Yes	15.4% (*n* = 38)	53.0% (*n* = 131)	31.60% (*n* = 78)	0.043	0.663
	No	15.0% (*n* = 15)	58.0% (*n* = 58)	27.00% (*n* = 27)		
Having and using the bicycle	Yes	15.3% (*n* = 40)	51.5% (*n* = 135)	33.20% (*n* = 87)	0.117	0.092
	No	15.3% (*n* = 13)	63.5% (*n* = 54)	22.20% (*n* = 18)		

Legend: Values are expressed as percentages (%) and absolute frequencies (*n*). Differences between categories were analysed using chi-square tests. * *p* ≤ 0.05. Abbreviations: Sig. = significance (two-tailed).

**Table 6:** MD Adherence by Satisfaction with PE.

	MD Adherence								*F*	*η* ^2^	Sig.
	Low-Quality Diet		Needs Improvement		Optimal Diet		Total				
	*M*	*SD*	*M*	*SD*	*M*	*SD*	*M*	*SD*			
**PE Overall Satisfaction**	5.68	1.07	6.09	1.21	6.38	1.18	6.11	1.20	6.267	0.035	0.002 ***
**Teaching**	5.56	1.45	6.21	1.42	6.45	1.44	6.18	1.46	6.949	0.039	0.001 ***
**Relaxation**	5.39	1.50	5.87	1.52	6.37	1.29	5.95	1.48	8.529	0.047	0.000 ***
**Cognitive development**	5.64	1.61	6.33	1.44	6.62	1.40	6.31	1.48	7.832	0.044	0.000 ***
**Health and Fitness** **Improvement**	5.67	1.35	6.15	1.48	6.41	1.41	6.16	1.45	4.734	0.027	0.009 ***
**Interaction with Others**	5.86	1.39	5.98	1.50	6.36	1.43	6.08	1.47	2.887	0.017	0.057
**Normative Success**	5.79	1.55	5.59	1.67	5.62	1.74	5.63	1.67	0.279	0.002	0.757
**Fun and Enjoyment**	5.79	1.49	6.20	1.71	6.50	1.60	6.23	1.66	3.290	0.019	0.038 ***
**Mastery Experiences**	5.89	1.58	6.15	1.61	6.58	1.41	6.24	1.56	4.134	0.023	0.017 ***
**Recreational Experiences**	5.65	1.24	6.06	1.37	6.36	1.46	6.09	1.39	4.717	0.027	0.010 ***

Legend: Differences between MD adherence groups (Low-quality diet, needs improvement, Optimal diet) were analysed using one-way ANOVA, with *F* and corresponding significance (Sig.) reported for each dimension. *** *p* ≤ 0.05. Abbreviations: Sig. = significance (two-tailed). *M* = Media; *SD* = Standard deviation.

## Data Availability

The original contributions presented in this study are included in the article. Further inquiries can be directed to the corresponding author.
